# Revision for the Rapid Emergency Triage and Treatment System Adult (RETTS-A) needed?

**DOI:** 10.1186/s13049-016-0254-z

**Published:** 2016-04-26

**Authors:** Amir Mirhaghi, Michael Christ

**Affiliations:** Evidence-Based Caring Research Center, Department of Medical-Surgical Nursing, School of Nursing & Midwifery, Mashhad University of Medical Sciences, Chahrrahe-Doktorha, 9137913199 Mashhad, Khorasan Razavi Iran; Universitätsklinik für Notfall- und Internistische Intensivmedizin, Paracelsus Medizinische Privatuniversität, Nürnberg, Klinikum Nürnberg Germany

## Abstract

The study highlights the prognostic role of patient’s vital signs at presentation to the emergency department (ED): The predictive role of vital signs in ED triage has been controversially discussed probably due to a paucity of data on the value of vital signs in ED at presentation. However, the authors did not find a suitable way to adjust for the inherent influence of triage decision and medical treatment on mortality. We have discussed that ambiguity concerning the assessment of vital signs criteria in RETTS-A Red priority may threaten any association between patient acuity and fatal outcome.

## Dear Editor

We have read the recent publication from Ljunggren et al. entitled “*The association between vital signs and mortality in a retrospective cohort study of an unselected emergency department population*” in *Scandinavian Journal of Trauma, Resuscitation and Emergency Medicine* with great interest [1].

The study highlights the prognostic role of patient’s vital signs at presentation to the emergency department (ED): The predictive role of vital signs in ED triage has been controversially discussed probably due to a paucity of data on the value of vital signs in ED at presentation [1] and the publication of small and/or selected cohorts [2].

In a large, unselected population, Ljunggren et al. convincingly show that the higher the deviation of vital signs from normal range is, the higher the odds of mortality are within 1 day and 30 days of follow-up. However, the authors did not find a suitable way to adjust for the inherent influence of triage decision and medical treatment on mortality. In addition, deviations of vital signs indicate different odds of mortality depending on chief complaints of patients at presentation.

Possibly, ambiguity concerning the assessment of vital signs criteria in RETTS-A Red priority may threaten any association between patient acuity and fatal outcome.

**Fig. 1:**
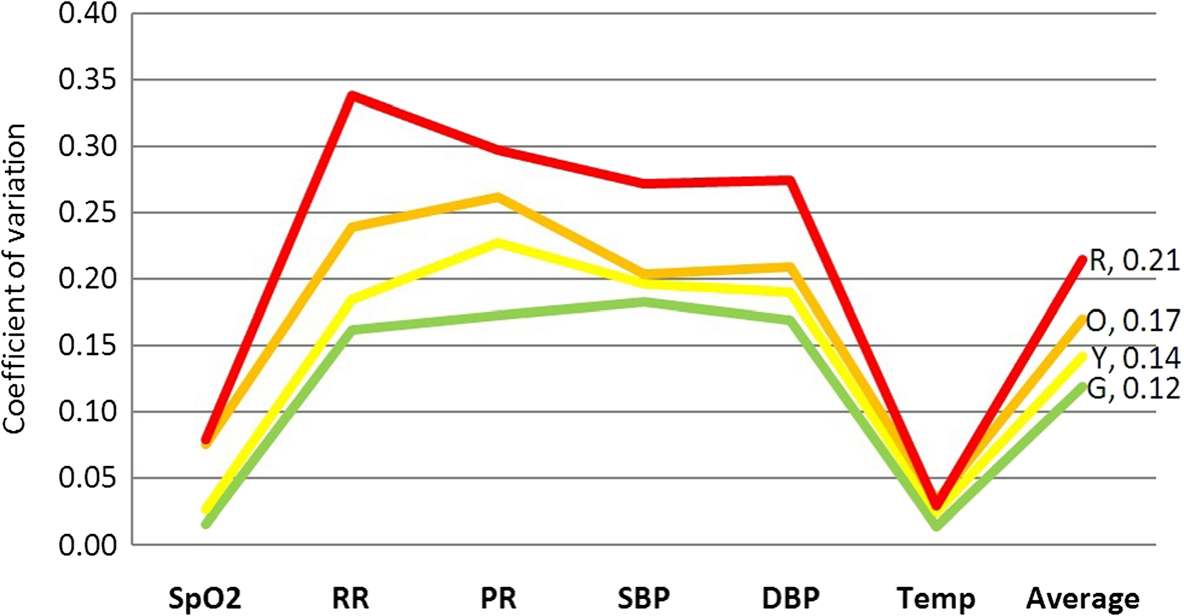
Coefficient of variation regarding vital signs

Four percent of patients have been allocated to the Red priority of RETTS-A triage system. This is considerably higher than the average patients’ number in the immediate level of other triage scales (2 % for level 1 five-point triage scale) [3]. It may be hypothesized that criteria for Red priority in RETTS-A are too broad leading to overtriage (4 % for Red priority). Our interpretation is supported by wide dispersion of vital sign measures in RETTS-Red priority of the current study. Among triage priorities, the highest coefficient of variation (CV) for vital signs occurred in the Red priority (0.21). CVs for Red, Orange, Yellow and Green priorities were 0.21, 0.17, 0.14 and 0.12 respectively (Fig. 1).

We would that CVs for Red and Green priorities would be lowest among RETTS-A priorities because the most and the least acuity patients usually are widely recognized as the most easily distinguishable subsets of patients in the ED [4, 5]. CV of 0.21 for Red priority indicates that this priority has been dispersed by high risk patients who may have a potential major life or organ threat instead of patients who are physiologically unstable and require immediate interventions. This ambiguity may result in a significant obstacle to the delivery of timely care for critically-ill patients [6]. It’s worth mentioning that standard deviation of all vital signs criteria in Red priority overlap with means of vital signs criteria of Green priority except respiratory rate. This indicates that stable patients in Green priority display comparable deviations of vital signs than critically-ill patients in Red priority.

It is tempting to speculate whether construct validity of RETTS-A may be improved by developing measures of cohesive, homogeneous entities for each priority [7]. Priority Red could be divided into two heterogeneous priorities including immediate and emergent priorities, resulting in 5-point RETTS. This may strengthen the association between fatal outcome and RETTS priorities. Possibly, a revision of RETTS-A triage system may help to further improve effectiveness.

## References

[1] Ljunggren M, Castrén M, Nordberg M, Kurland L (2016). The association between vital signs and mortality in a retrospective cohort study of an unselected emergency department population. Scand J Trauma Resusc Emerg Med.

[2] Cooper RJ, Schriger DL, Flaherty HL, Lin EJ, Hubbell KA (2002). Effect of vital signs on triage decisions. Ann Emerg Med.

[3] Hing E, Bhuiya FA (2012). Wait time for treatment in hospital emergency departments: 2009.

[4] Mirhaghi A, Heydari A, Mazlom R, Ebrahimi M (2015). The reliability of the Canadian triage and acuity scale: meta-analysis. N Am J Med Sci.

[5] Mirhaghi A, Kooshiar H, Esmaeili H, Ebrahimi M (2015). Outcomes for emergency severity index triage implementation in the emergency department. J Clin Diagn Res.

[6] Christ M, Grossmann F, Winter D, Bingisser R, Platz E (2010). Modern triage in the emergency department. Dtsch Arztebl Int..

[7] Smith GT, McCarthy DM, Zapolski TC (2009). On the value of homogeneous constructs for construct validation, theory testing, and the description of psychopathology. Psychol Assess.

